# Physicochemical and Functional Characterization of Female Reproductive Fluids: A Report of the First Two Infants Born Following Addition of Their Mother's Fluids to the Embryo Culture Media

**DOI:** 10.3389/fphys.2021.710887

**Published:** 2021-09-01

**Authors:** Analuce Canha-Gouveia, Maria Teresa Prieto-Sánchez, Maria Luisa Sánchez-Ferrer, Marta Mollá, Juan Carlos Martínez-Soto, Evelyne París-Oller, Cristina Soriano-Úbeda, José Landeras, Pilar Coy

**Affiliations:** ^1^Department of Physiology, Faculty of Veterinary, University of Murcia, Murcia, Spain; ^2^Institute for Biomedical Research of Murcia IMIB-Arrixaca, Murcia, Spain; ^3^Department of Obstetrics and Gynecology, “Virgen de la Arrixaca” University Clinical Hospital, Murcia, Spain; ^4^IVI Murcia, Murcia, Spain

**Keywords:** human female reproductive fluids, embryo culture media, quality control sheet, osmolality, pH, protein concentration

## Abstract

Culture media supplemented with reproductive fluids (RF) have been used in livestock species, improving the efficiency and quality of *in vitro* produced embryos. However, usefulness in humans is still unknown. In this study, we collected human reproductive fluids (HRFs) *ex vivo* (from 25 patients undergoing abdominal hysterectomy plus bilateral salpingectomy) and *in vivo* (from 31 oocyte donors). Afterward, protocols to evaluate their osmolality, pH, total protein concentration, endotoxin level, and sterility were optimized, establishing security ranges for their use as natural additives. In addition, a functional assay was developed with bovine embryos grown in vitro in a medium supplemented with 1% of collected HRFs. Finally, a proof of concept was performed with six patients on post ovulation day 2 to evaluate the full-term viability of embryos grown in media supplemented with autologous uterine fluid, collected under *in vivo* conditions. Two of the embryos resulted in successful pregnancy and delivery of healthy babies. In conclusion, this study establishes a complete quality control sheet of HRFs as additives for embryo culture media and shows first preliminary data on obtaining healthy offspring derived from embryos grown in media supplemented with HRFs.

## Introduction

Female reproductive fluids (RFs) are physiological “culture media” where first events in the life cycle take place: oocytes grow and mature inside the follicular fluid; then, a mature oocyte is fertilized by sperm in the oviduct, where gametes are surrounded by the oviductal fluid (Avilés et al., 2010). After fertilization and first cell divisions, the embryo moves toward the uterus around days 3–5 post-fertilization, where endometrial glands secrete uterine fluid, which, is essential for embryo implantation and survival (Zhang et al., 2017). These RFs have been widely studied to identify novel markers of endometrial receptivity and gain significant insights into the mechanisms underlying unexplained infertility, recurrent pregnancy losses, and other endometrial pathologies (Salamonsen et al., 2013; Bhusane et al., 2016). Furthermore, these RFs have been proposed to supplement artificial media used in assisted reproductive technologies (ARTs), since their addition could mimic the natural environment of the female tract and could minimize the stress in gametes and embryos caused by artificial composition media, which have been linked to a possible greater risk of short- and long-term health problems of the offspring (Adamson et al., 2011; El Hajj and Haaf, 2013). Although not yet tested in humans, RFs have been successfully used as a natural additive in two major livestock species, the pig and the cow, resulting in the birth of healthy offspring (Lopes et al., 2020; Paris-Oller et al., 2021). Moreover, the addition of RFs in small volumes (<2% of fluid) in these mammalian models promoted improvements in terms of the number of cells per blastocyst, developmental kinetics of the embryos, cryo-tolerance, and DNA methylation and gene expression patterns (Lopera-Vasquez et al., 2015; Canovas et al., 2017). However, RFs are potentially problematic in terms of biosecurity and reproducibility of results, and their availability is scarce. Therefore, before using HRFs as additives in future clinical trials on humans, it is necessary to standardize an effective collection method and adequate population of donors and establish a biobank collection of these fluids fulfilling all sanitary and legal requirements. Furthermore, to guarantee that their addition is made under strict conditions of biosecurity and that they are beneficial to the embryo, it is important to evaluate if the collected fluids have compatible physicochemical properties with the ones recommended for the commercial media usually used in infertility clinics. Usually, the quality sheet of most commercial culture media for gamete collection, fertilization, and embryo culture presents as release specifications, pH (in the air) values between 7.3 and 7.8, osmolality values between 285 and 295 mOsm/kg, human serum albumin values of 2–12.5 mg/ml, two-cell mouse embryo assay (MEA) ≥ 80% of control expanded to blastocysts at 72 h, endotoxin levels below 0.4 EU/ml, and aseptically filtration as a sterilization method. It is known that several factors, such as oxygen, temperature, pH, osmolality, and molecular composition of culture media, may influence the morphology, developmental kinetics, epigenetic profile, physiology, and metabolism of human embryos (Gardner and Kelley, 2017). Consequently, it is crucial to establish security ranges for all these parameters in HRFs, especially human oviductal fluids (HOFs) and human uterine fluids (HUFs) in which gametes and embryos develop naturally.

The collection of HRF has already been performed previously by uterine flushing and aspiration for different purposes (Hannan et al., 2012). Usually, embryo transfer catheters connected to a syringe with enough volume (10–20 ml) to perform aspiration or uterine flushing by manual vacuum application are used (Ametzazurra et al., 2009). Cervical mucus aspiration catheters can also be used to collect HRFs, avoiding uterine damage and taking advantage of the integrated plunger to perform a more efficient aspiration (Canha-Gouveia et al., 2019).

The collection of HRF has been reported previously in healthy patients by *ex vivo* aspiration of their fallopian tubes, as well as by *in vivo* aspiration of the uterine cavity (Canha-Gouveia et al., 2019). However, it is known that there is a loss of fertility with aging, mainly due to the decrease in the quantity and quality of the gametes (Baird et al., 2005). This decrease is more noticeable from the mid to late 30s and more profound afterward until menopause (Steiner and Jukic, 2016). Therefore, since the age of the women participating in previous study (Canha-Gouveia et al., 2019) was above 30 years old, it is important, besides studying the physicochemical and functional properties of HOFs in these women, to compare them with younger women such as those who are oocytes donors attending fertility clinics, and under *in vivo* conditions. Unfortunately, while the *in vivo* collection of HUFs is feasible in the young healthy women population, the same is not possible for HOFs because of the risk of irreversible damage during manipulation of the fallopian tubes. Therefore, only *ex vivo* samples of this fluid can be studied.

Regarding biosecurity, it is also necessary to assure that HRF does not contain pathogenic agents that could represent a risk of disease transmission. To this end, protocols for quantifying the endotoxin levels in HRFs and evaluating environmental microbiological contamination during their collection and storage must be developed. Culture media are usually sold as “endotoxin-free” or certified with endotoxin levels ≤ 0.1 EU/ml by *Limulus* amebocyte lysate (LAL) test (Bang, 1956; Levin and Bang, 1964, 1968). Since there is no information on the endotoxin ranges of HRFs, it is imperative to establish suitable methods to perform the quantification. Similarly, exhaustive controls must be performed on the process of HRF collection and storage to guarantee the lack of environmental contamination. Standardized protocols for this purpose remain to be defined.

This study aimed to (1) establish and optimize the mentioned protocols, using as little volume as possible, to define the physicochemical properties (osmolality, pH, and total protein concentrations) of HUFs collected under different conditions (HUF *in vivo* vs. HUF *ex vivo*) and HOFs collected *ex vivo*; (2) to evaluate the functional properties (embryo assay) of HRFs, with sterile and endotoxin-free HUFs collected under *in vivo* conditions; (3) finally, to develop a preliminary proof of concept by transferring embryos cultured with a small proportion of HUFs collected from their mothers.

## Materials and Methods

### Ethics Statement

This study was approved by the Ethics Research Committee (CEIC) of Clinical University Hospital “Virgen de la Arrixaca” (HCUVA), Murcia, Spain (Approval No. EST: 04/16). All the women who accepted to participate in this study provided their written informed consent. The proof of concept “Validation of Addition of Uterine Fluid to Human Embryo Culture Medium”−1607-MUR-055-JL/ was approved on 08/21/2019 and registered on ClinicalTrials.gov of the United States National Library of Medicine with the identifier NCT03436758. All the human RFs were collected following standard operating procedures in accordance with Directive 2004/23/EC of the European Parliament and of the Council of March 31, 2004, concerning human blood and its components, Law 14/2007, of July 3, of Biomedical Research, and Royal Decree of Biobanks 1716/2011, of November 18.

### Physicochemical and Functional Characterization of HRF

#### Study Population

##### Group A: *Ex vivo* Oviductal and Uterine Fluid Collections From Women Undergoing Gynecology Surgery

The *ex vivo* collections were performed in women under 50 years old who underwent abdominal hysterectomy plus bilateral salpingectomy due to benign uterine pathology. The surgeries were performed at the Service of Obstetrics and Gynecology of the HCUVA from January 2016 to June 2018 by two experts and trained gynecologists. All the women included in the study had not undergone hormone treatment during the 3 months before surgery, had no fertility problems in their medical history, endometriosis, or any other adnexal pathology, which was ruled out after the histological study.

##### Group B: Oocyte Donors—*in vivo* Uterine Fluid Collections

The *in vivo* collection was performed in women under 35 years old participating in the oocyte donation program at the IVI-RMA Murcia clinic, between January 2017 and January 2018. All of them were previously accepted for the donation bank according to criteria established in the IVI-RMA clinic, demonstrated proven fertility, and accepted to have a sample of HUFs collected. All the donors were subjected to perceptive serological controls applied routinely in these cases, such as Hepatitis B HBs Ag (surface antigen), Hepatitis C HCV Ac (total antibodies), HIV Ac/Ag (antigen and antibodies), Treponema pallidum Ac (antibodies), and Hepatitis B HBc (core) Ac (core antibody).

#### HRF Collection

On the one hand, the oviductal fluid (HOF *ex vivo*) was collected exclusively *ex vivo* from the fallopian tubes after the internal genitalia were removed by abdominal surgery. Once dissected, the fallopian tubes were clamped in both extremities to avoid sample waste. With an ascendant manual mechanical pressure between the extremities, the oviduct fluid that accumulated at the upper portion of the ampulla was aspirated through the Mucat device. On the other hand, the non-diluted HUF was obtained under *ex vivo* conditions from the patients who underwent a planned abdominal hysterectomy (HUF *ex vivo*) and under *in vivo* conditions from oocyte donors after transvaginal oocyte retrieval, with an adapted Mucat device (CDD Laboratories, San Antonio, TX, United States). Once this device was introduced into the uterus, aspiration of the fluid was performed with the integrated plunger as described previously by Canha-Gouveia et al. (2019). The HRF collection was performed by the same two researchers in all the surgeries. All the samples were collected in EDTA K2 (1.8 mg/ml) tubes. The samples that presented evidence of blood contamination were discharged. The others were immediately centrifuged at 7,000 *g* for 15 min at 4°C to remove cell debris, and the supernatant was aliquoted and frozen at −80°C until analysis. All the collected samples were stored at Biobank-Mur (Biobancoen Red de la Región de Murcia, PT13/0010/0018; PT17/0015/0038, integrated into the Spanish National Biobanks Network, B.000859). A scarce volume of fluid was achieved with the method described previously, either *in vivo* or *ex vivo* (13.9–96.3μl for HUFs and 8.9–38.1 μl for HOFs) (Table 1). Therefore, it became necessary to optimize the protocols to establish corresponding security ranges using the minimum effective volume.

**Table 1:** Demographic characteristics of the study population and collected volume (mean ± SD) of reproductive fluids (RFs) for physicochemical and functional characterization.

		**GROUP A**	**GROUP B**
		**Obstetrics/Gynecology Patients**	**Oocyte donors**
	Total N	25	31
	Age (years)	45 ± 4	25 ± 4
	HRF Collection	*Ex vivo*(mucat device)	*in vivo*(mucat device)
HUF	Total (n)	20	31
	Volume (μL)	62.8 ± 33.5	40.7 ± 26.8
HOF	Total (n)	25	–
	Volume (μL)	23.5 ± 14.6	–

*HRFs, human reproductive fluids; HUF, human uterine fluids; HOFs, human oviductal fluid*.

#### Osmolality

To evaluate osmolality, the minimum volume needed was set at 10 μl. Quantification was performed using a Vapro® 5520 (Wescor Inc., Logan, Utah, USA). This type of osmometer allowed the determination of osmolality in mOsm/kg by ebullioscopy, which is suitable for micro samples. Osmolality was determined in 17 samples of HUFs from the oocyte donors (*in vivo* group) and in 9 and 10 samples, respectively, of HOFs and HUFs from the patients (*ex vivo* group). In addition, the osmolality of the six samples of diluted HUFs from the embryo recipients was also determined.

#### pH

Most of the equipment used to measure the pH requires a minimum volume of 100 μl, which is not usually reached during the collection of RF. To reduce the volume of sample used and obtain a more accurate measurement, a specific electrode for micro samples was used (“pH OxyMini”; PreSens, Regensburg, Germany) (Albors et al., 2020). To evaluate the pH (in the air) of the collected samples, enough volume of each specimen (20–30 μl) to adequately cover the electrode was put in Eppendorf tubes on stable support placed on ice to avoid the influence of temperature variation on the accuracy of pH measurements. The probe was introduced into the tube, the pH was measured, and a graph with the values was made. The pH value that remained stable after 2–4 min was taken as valid. The pH was determined in the 17 samples of HUFs from the oocyte donors (*in vivo* group) and in the 12 samples of HOF and HUF from the patients (*ex vivo* group). In addition, the pH of the six samples of diluted HUF from the embryo recipients was determined to confirm that it was at established security levels.

#### Total Protein Concentration

For the quantification of total proteins, the commercial kit Coomassie (Bradford) Protein Assay Kit (Thermo Fisher Scientific, Waltham, MA, United States) (Cat. No 23200) was used. Each sample was diluted in water (5μl of RF in 95 μl of water). Aliquots of 30 μl of each diluted sample were prepared in 1.5 ml of the Coomassie reagent (Coomassie dye G-250, methanol, phosphoric acid, and water). After gentle mixing, the solution was incubated at room temperature for 5 min. Then, the samples were transferred to cuvettes that were introduced in a spectrophotometer capable of measuring absorbance at 595 nm. The curves obtained were compared with previously prepared standard curves using standard bovine serum albumin (BSA) protein. A total of 9 samples of HOFs and 15 samples of HUFs from the oocyte donors (*in vivo* group) and 6 samples of diluted HUFs from the embryo recipients were assessed with this method.

#### Hemoglobin Concentration

For the measurement of this parameter, a HemoCue® (HemoCue AB, Ängelholm, Sweden) Plasma/Low Hb photometer was used. The first step was thawing of the samples, after which controls were used to calibrate the apparatus. Once the samples were thawed, 10 μl of each were transferred into microcuvettes. Each microcuvette was introduced inside the photometer for reading, with the result appearing on the screen after 10 s. A total of 10 HUF and 9 HOF samples were assessed with this method.

#### Sterility

Samples of reproductive fluids were diluted to 1% sterile saline and analyzed with a BACT/ALERT® (Biomérieux Inc., Durham, NC, USA) three-dimensional microbial detection system after 7 days of incubation. A total of 15 samples of HUF from the oocyte donors (*in vivo* group) and 6 samples of diluted HUFs from the embryo recipients were assessed for sterility conditions.

#### Endotoxin Quantification

Endotoxin quantification was performed using the Endosafe® kit (Charles Rivers®, Ecully, France), with a maximum sensitivity of 0.005 EU/ml. This test is routinely used in human plasma samples but not in HRF, and it was necessary to calibrate the apparatus with different dilutions and to ask for advice from the company (Charles River®, Ecully, France) before establishing the final protocols. A total of 15 samples of HUFs from the oocyte donors (*in vivo* group) and 6 samples of diluted HUFs from the embryo recipients were assessed, according to the recommendations of the producer (Charles River Endosafe Ltd., Ecully, France).

### *In vitro* Culture of Bovine Embryos (Bovine Embryo Assay, BEA)

To characterize at a functional level the HRF as supplements for embryo culture media, a bovine embryo assay was established. *In vitro* maturation of bovine oocytes from ovaries obtained in a slaughterhouse was performed following the protocol described by Lopes et al. (2019). The COCs collected previously were washed two times in a maturation medium consisting of TCM-199 (with Earle's salts); supplemented with 26.2 mM sodium bicarbonate, 0.2mM sodium pyruvate, 2 mM glutamine, 50 mg/ml gentamycin, 10 IU/ml equine chorionic gonadotropin (Foligon; Intervet International BV, De Bilt, Netherlands), and 10 IU/ml human chorionic gonadotropin (VeterinCorion; Divasa-Farmavic, Barcelona, Spain); divided into groups of 30–50; and transferred to four-well dishes (cell culture tested; Nunc®, Roskilde, Denmark) in a total volume of 500 μl, with 10% (v/v) fetal bovine serum. About 30 min before IVF, the COCs were rinsed two times and transferred to a Fert-TALP medium supplemented with 2 mg/ml heparin and 50 mg/ml gentamycin. Frozen semen from two bulls with known fertility was used. Thawing was performed in a water bath at 38°C for 30 s, and the semen was transferred to a 15 ml Falcon tube with 10 ml Sperm-TALP medium supplemented with 50 mg/mL gentamycin and incubated at 38°C for 10 min. The sperm was centrifuged at room temperature at 700 × *g* for 3 min, and the supernatant was discarded. Sperm motility and concentration were assessed, and the sperm was diluted in Fert-TALP prior to insemination at a final concentration of 1 million spermatozoa per milliliter. IVF took place at 38.5°C in a humidified atmosphere with 5% CO_2_. About 18 to 20 h post-insemination (hpi), potential zygotes were put in a 15 ml Falcon tube with 2 ml of Dulbecco's phosphate-buffered saline (DPBS) and vortexed for 3 min. Presumptive zygotes were washed two times in DPBS and three times in an embryo culture medium (synthetic oviduct fluid, SOF, Holms) supplemented with BSA before transferring them to 25 ml micro drops of the embryo culture medium containing (experimental group) or not (control group) 1% of HUF, covered with paraffin oil (NidOil™; Nidacon, Molndal, Sweden), with a maximum load of 25 embryos/micro drop. Incubation took place at 38.5°C, 5% CO_2_, and 5% O_2_ for 48 h. After *in vitro* culture, the proportion of presumed zygotes that reached the two-cell stage was quantified. In bovine species, the average percentage of bovine embryos at the two-cell stage on day 2 of embryo culture is around 70% and the percentage of embryos expanded to blastocyst is close to 30% (Lonergan and Fair, 2014). Therefore, these values were established as a cut-off to approve the sample. A total of 14 samples of HUFs from the oocyte donors (*in vivo* group) and six samples of diluted HUFs from the embryo recipients were assessed by BEA.

### Proof-of-Concept “Validation of Addition of Uterine Fluid to Human Embryo Culture Medium”−1607-MUR-055-JL/ NCT03436758

#### Study Population

The proof of concept to evaluate the feasibility of the supplementation of embryo culture media with HRF was performed only with HUF since HOF cannot be obtained in these patients as explained previously. Furthermore, the influence of the constituents of HRF in embryo culture media could be evaluated mainly with the addition of HUF, since both fluids are very similar in women in terms of protein composition (Canha-Gouveia et al., 2019). All couples attending the IVI Murcia clinic undergoing IVF treatment and ICSI, from January 2017 to December 2019, who meet the following inclusion criteria were invited to participate in this proof-of-concept for women, of normal weight, younger than 39 years old, retrieval of at least eight metaphase II oocytes, and transfer of their own embryos; for men, sperm concentration > 5 million/ml. The exclusion criteria for women were diagnosis of endometriosis or uterine pathology and patients who underwent IVF treatment for repeated abortion or implantation failure, abnormal karyotype or included in the Pre-implantation Genetic Diagnosis (PGD) program. The exclusion criteria for men were abnormal karyotype, high values of DNA fragmentation, pathological fluorescence *in situ* hybridization (FISH), and sperm samples obtained from epididymal aspirate or testicular biopsy.

#### Superovulation of Proof-of-Concept Participants

The participating women underwent a controlled ovarian stimulation with follicle-stimulating hormone (FSH) at daily subcutaneous doses of 150–225 IU/ml to generate follicular development. When the follicular size reached 13–14 mm, it was added to the stimulation protocol, gonadotropin-releasing hormone (GnRH) antagonist (Orgalutrán) 0.25 mg of Ganirelix subcutaneously, to avoid the endogenous surge of LH because of the increase in serum estradiol level. Once the adequate follicular size was achieved (follicular diameter > 18 mm), both the FSH and the GnRH antagonist were interrupted, and final oocyte maturation was induced with a GnRH agonist analog (Decapeptyl) 0.2 mg subcutaneous of bolus triptorelin 36 h before scheduling the transvaginal ultrasound-guided ovarian puncture to perform oocyte retrieval. This periovulatory HUF was collected by uterine flushing, introducing an “embryo-transfer” cannula (Delphin cannula; Gynetics Medical Products, Lommel, Belgium) connected to a 10 ml syringe into the uterine cavity with 400 μl of embryo culture media (Gems Geri® medium; Genea Biomedx, Sydney, Australia). Uterine flushing was performed by manual vacuum aspiration with the syringe. Only the samples of diluted fluid derived from uterine flushing showing no presence of blood were considered for analysis. Samples were expelled into standard cryogenic tubes and immediately frozen at −80°C until processed. HUF collected samples were also centrifuged at 7,000 *g* for 15 min at 4°C to remove cell debris, and the supernatant was aliquoted and frozen at −80°C. Afterward, each HUF collected was assessed in terms of osmolality (mOsm/kg), pH, total protein (μg/μl), sterility, endotoxin (EU/ml), and BEA (bovine embryo assay, two cells). Only the samples that fit the security levels stated in the QC data sheet previously established were validated to be used in the proof of concept. The percentage of fluid that should be used in this proof of concept was established after five previous quantifications of the albumin concentration in pure vs. diluted HUF, in all of them, it was shown that the proportion of albumin was reduced ~10 times, so 10% of diluted HUF should, theoretically, be equivalent to 1% of pure HUF. This proportion of 1% is what has been used in the experiments performed with animal models (Canovas et al., 2017; Lopera-Vasquez et al., 2017).

#### Oocyte Retrieval and Embryo Culture (Control Media vs. Media Supplemented With Autologous HUF Collected Samples)

At the day of the transvaginal ultrasound-guided ovarian puncture, oocytes of the six patients who fulfilled all the inclusion and exclusion criteria, and whose human uterine fluid samples matched the QC datasheet, were efficiently retrieved and washed in a Sequential FertTM medium. The removal of cumulus cells was performed by gently pipetting the oocytes in a solution of 80 IU/ml hyaluronidase in a Sequential FertTM medium. The oocytes were cultured in a fertilization medium (GemsR; GeneaBiomedx, Sydney, NSW, Australia) at 5% CO_2_, 37°C, and atmospheric O_2_ for 3 h. After that, the oocytes were placed in a micro drop of fertilization medium for performing intracytoplasmic sperm injection (ICSI). Sperm samples were obtained from ejaculates without oligo-asteno-teratozoospermia and selected by density gradient 45/90% (SIP100; Sil-Select Plus, FertiPro, Beernem, Belgium), diluted in a solution of 10% polyvinylpyrrolidone (PVP) in the Sequential FertTM medium, and placed in a micro drop for performing ICSI. All the patients underwent ICSI to reduce the biases derived by the technique used for fertilization as well as to obtain the highest number of embryos. Immediately after ICSI, half of the zygotes were transferred to the pre-equilibrated embryo culture medium Cleavage MediumTM (GemsR, GeneaBiomedx, Sydney, NSW, Australia) and the other half was cultured in the same media supplemented with 10% diluted uterine fluid of the mother (autologous culture), and all of them were covered with mineral oil (LifeGuardR; Genomicks SdnBhd, Petaling Jaya, Malaysia) and cultured for 5 days. The embryonic quality and kinetics of each embryo were evaluated blindly by morphological scoring on day 5 of culture (blastocyst stage) by the same embryologist according to the standardized criteria of the Spanish Association for the Study of the Biology of Reproduction (ASEBIR) (Cuevas Saiz et al., 2018; Staicu et al., 2021). Only the best-quality embryo according to morphological criteria was transferred to the respective receptor. The number of embryos successfully implanted and the number and sex of newborns were registered.

### Statistical Analysis

The normality of the data was tested by the Shapiro–Wilk test. According to this, a 99 or 95% CI for the mean was chosen to set reference values. To compare parameters between the three groups (HUF *in vivo*, HUF *ex vivo*, and HOF), Student's *t*-tests were performed because of the normality and independence of the data. For the samples HUF *ex vivo* and HOF *ex vivo* collected from the same donors, a paired-sample *t*-test was performed. For the samples collected from different women, an independent sample *t*-test was performed. Differences were considered significant when *p* < 0.05 or *p* < 0.01.

## Results

### Establishment of a QC Sheet for Collected HRFs

The quality control (QC) sheet for HRF collected *in vivo* and *ex vivo* to be used as new natural products in culture media was achieved by the characterization of non-diluted HOFs and HUFs in patients and donors. The *ex vivo* collection of HUFs and HOFs was successfully performed in 25 patients with an average age of 45 years, and *in vivo* collection (HUFs) was also successfully performed in 31 oocyte donors with an average age of 25 years (Table 1). Because of the variability in the volume of fluid collected per woman, it was not possible to check all the parameters in all the samples (Table 1). The volume of fluid collected from the participants of the proof of concept is not included in Table 1, because it was collected by flushing instead of being pure.

### Osmolality

**Figure 1:**
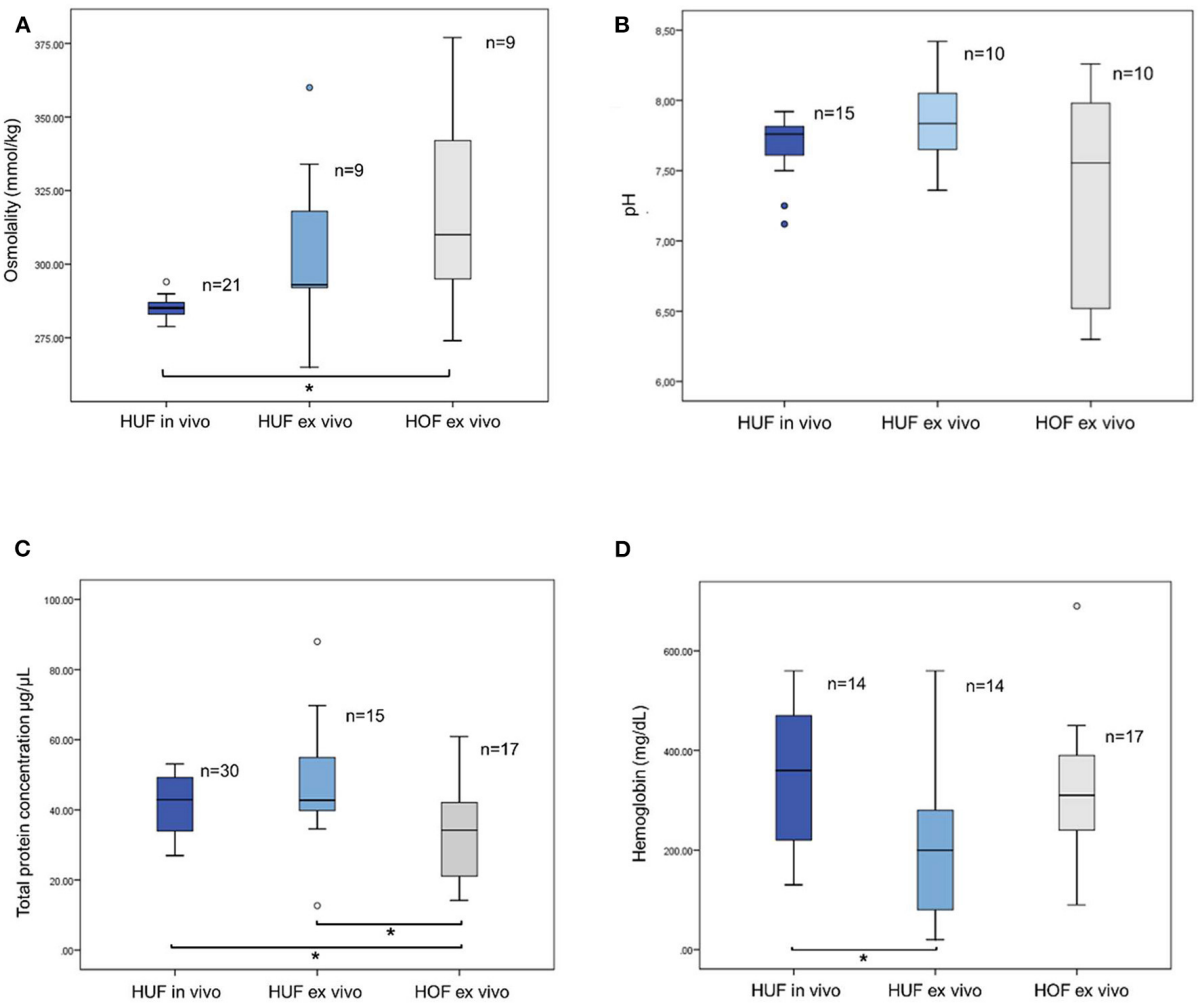
**(A)** Osmolality values for human uterine fluids (HUFs) and human oviductal fluids (HOFs). The uterine fluids were collected either from oocyte donors (HUFs *in vivo*) or from women undergoing hysterectomy (HUF *ex vivo*). Oviductal fluids were collected from women undergoing salpingectomy (HOF *ex vivo*). ^*^ Indicates significant differences (*p* < 0.05). **(B)** pH values for human uterine fluids collected *in vivo* (HUF *in vivo*), or *ex vivo* (HUF *ex vivo*), and human oviductal fluids collected *ex vivo* (HOF *ex vivo*). The two blue dots below the HUF *in vivo* bar correspond to the values of the outliers. No significant differences (*p* < 0.05) were found among the three groups. **(C)** Total protein concentration for human uterine fluid collected *ex vivo* (HUF *ex vivo*) and for human oviductal fluids collected *ex vivo* (HOF *ex vivo*). ^*^Indicates significant differences (*p* < 0.05). **(D)** Hemoglobin concentration for human uterine fluids collected *ex vivo* (HUF *ex vivo*) and for human oviductal fluids collected *ex vivo* (HOF *ex vivo*). ^*^Indicates significant differences (*p* < 0.05).

The osmolality values of the HUF samples from oocyte donors (HUFs *in vivo* group) conditions ranged between 278.8 and 294 mOsm/kg (Figure 1A). The mean value (±SD) was 285.21 (±0.77). The values from HUF samples collected from women undergoing hysterectomy (HUF *ex vivo*) ranged between 265 and 360 mOsm/kg, and the mean ± SD was 304.67 ± 9.79 (Figure 1A). Finally, the values for HOF ranged between 274 and 377, with a mean ± SD of 316.56 ± 12.33 (Figure 1A).

According to these data, values within 95% CI for the mean were chosen as the criterion to select the physiological ranges, so it was decided to set the acceptable osmolality for HUFs between 284 and 287 and 282 and 327 mOsm/kg for the *in vivo* and *ex vivo* groups, respectively. As for HOFs, and following the same criterion, the range was established between 288 and 344 mOsm/kg. All analyzed samples found within that range were considered suitable for use as a future supplement in embryo culture.

### pH

The values of pH in HUF from the *in vivo* group ranged from 7.12 to 7.92, with a mean ± SD value of 7.7 ± 0.23 (Figure 1B). Because of the normality properties of the data, values within the 99% CI for the mean were chosen to be included as physiological ranges, so samples with values between 7.46 and 7.94 were considered suitable for ART use. The values for HUF from the *ex vivo* group ranged between 7.51 and 8.16 (Figure 1B), with the results of mean ± SD being 7.84 ± 0.32b. Following the same criterion, the physiological ranges included were those between 7.6 and 8.2. Finally, the values for HOFs were found between 6.3 and 8.36, with the physiological range being established between 6.62 and 8.12 (Figure 1B).

### Total Protein Concentration

The total protein concentration of HUF samples from oocyte donors (HUFs *in vivo* group) ranged between 26.97 and 53.1 μg/μl (Figure 1C). The mean ± SD was 41.9 ± 1.47 μg/μl. The values for the HUF *ex vivo* group ranged between 12.68 and 87.95 μg/μl, with the mean ± SD being 47.5 ± 4.5 (Figure 1C). Finally, the values for HOFs ranged between 14.18 and 60.9, with a mean ± SD of 32.21 ± 3.11 (Figure 1C). According to the values within the 95% CI for the mean, the values for the QC certificates were established between 38.9 and 44.9 μg/μl for HUF *in vivo*, 37.8 and 57.1 μg/μl for HUF *ex vivo*, and 25.6 and 38.8 μg/μl for HOF *ex vivo* (Figure 1C).

### Hemoglobin Concentration

As described above, there was significant differences between total protein concentration of HOF and HUF, both *in vivo* and *ex vivo* (*P* < 0.05). If such differences were an intrinsic characteristic of each reproductive fluids, or if they were the consequence of a lower blood contamination of oviductal samples it was a hypothesis that we wanted to test. Therefore, we also quantified the haemoglobin of these fluids to evaluate if this was the main cause of those differences. As we can see in the (Figure 1D), there were not significant differences in terms of hemoglobin concentration between uterine fluid and oviductal fluid. Therefore, we discarded the hypothesis that oviductal fluid had a lower total protein concentration due to less blood contamination. The hemoglobin concentration of HUF samples from oocyte donors (HUF, *in vivo* group) ranged between 268.60 mg/dL and 428.54 mg/dL (Figure 1D). The mean ± SD was 348.57 ± 37.02 mg/dL. The values from HUF samples collected from women undergoing hysterectomy (HUF *ex vivo*) ranged between 117.10 and 324.33 mg/dL, with the mean ±SD being 220.71 ± 47.96 (Figure 1D). Finally, the values for HOF samples collected from women undergoing hysterectomy ranged between 240.34 and 384.37 mg/dL, with a mean ±SD of 312.35 ± 33.97 mg/dL (Figure 1D).

### Sterility and Endotoxin Assessment

To evaluate the functional properties of the HRF, only the sterile and endotoxin-free samples were studied because any biological reagent used in humans should not contain pathogenic agents that could represent a risk of disease transmission. Consequently, endotoxins and sterility were evaluated first. Sterility controls were done only in the HUF samples collected *in vivo*, with the result in all of them being “no growth.” The criterion for this parameter had no ranges or options other than the absence of any growth of microorganisms to be considered as suitable for biobanking or use in ART.

Similarly, two samples were discarded because they did not fall in the range considered suitable for embryo culture media, according to the European Pharmacopoeia, and could not consequently be considered as “endotoxin-free” or certificated with endotoxin levels below ≤ 0.1 EU/ml.

### Bovine Embryo Assay (BEA)

The percentage of cleavage in bovine embryos *in vitro* fertilized and further cultured with 1% of HUFs added to the medium was above 70% for 13 of the 14 HUF samples tested *(in vivo* group) (Table 2). Eight days after *in vitro* fertilization (IVF), an average of 38 ± 18% of the cleaved bovine embryos reached the blastocyst stage (Table 2). The sample (number 5) with <70% of cleavage (47%) and the samples (10 and 14) with <30% embryos expanded to blastocyst (Lonergan and Fair, 2014) were considered not suitable for biobanking or use in ART, but the remaining 11 were considered suitable for both purposes.

**Table 2:** Percentage of cleavage (bovine cleaved embryos) and blastocyst rate (embryos that reached the blastocyst stage) after 8 days of culture with 1% of the respective HUF.

**HUF sample no**.	**COCs (Total N)**	**Cleavage**		**Blastocyst rate**	
		**Total N**	**%**	**Total N**	**%**
**1**	26	21	81	10	48
**2**	31	26	84	8	31
**3**	28	23	82	6	26
**4**	28	21	75	16	76
***5***	47	22	47*	
**6**	30	23	77	13	57
**7**	40	31	78	9	29
**8**	24	18	75	9	50
**9**	19	15	79	8	53
***10***	21	15	71	4	27*
**11**	30	22	73	9	41
**12**	28	20	71	6	30
**13**	34	24	71	10	42
***14***	22	17	77	5	29*

*The samples with <70% of cleavage or with <30% (asterisk) embryos reaching the blastocyst stage were considered not suitable for biobanking or use in ART, but the remaining 11 were considered suitable for both purposes*.

### QC Sheet for Human Reproductive Fluids Collected *in vivo* and *ex vivo*

The statistical analysis of the previous results allowed us to set reference values for each characteristic. Therefore, a complete QC sheet (Table 3) was developed for new potential natural additives, HOFs, and HUFs, which included security levels that should be taken into account regarding osmolality, pH, endotoxins, sterility, and total protein concentration, and for a functional assay using bovine gametes and embryos.

**Table 3:** Quality control sheet for HRFs collected *in vivo* and *ex vivo* to be used as new natural products in culture media.

	**HUF *IN VIVO***	**HUF *EX VIVO***	**HOF *EX VIVO***
Osmolality (mOsm/kg)	284–287	282–327	288–345
pH	7.5–7.9	7.5–8.2	6.6–8.1
Total protein (μg/μl)	39–45	38–57	26–39
Sterility	“no growth”		
Endotoxin (EU/ml)	“endotoxin-free” ≤ 0.1		
BEA (bovine embryo assay, 2 cell)	>70%		

*HRFs, human reproductive fluids; HUFs, human uterine fluids; HOF, human oviductal fluids*.

### Proof of Concept: Evaluation of Reproductive Fluids as Safe Additives for Embryo Culture Media in Humans

Fourteen women under 39 years of age were accepted to participate in the proof-of-concept NCT03436758QC, but only six fulfilled all the inclusion and exclusion criteria, namely, the retrieval of at least 8 metaphases II oocytes. All the six women had at least metaphase II oocytes, half of which were cultured with autologous 10% HUF of uterine flushing previously performed. All the collected samples were under the physiological ranges determined previously for the *in vivo* and *ex vivo* groups (Table 4A), and sterility and functional properties were assessed, by BEA, as adequate.

**Table 4A:** Physicochemical properties of HUF samples collected by uterine flushing of women who accepted to participate in the proof-of-concept NCT03436758QC and fulfilled all the inclusion criteria, namely, the retrieval of at least eight metaphase II oocytes and transfer of their embryos.

**Patient**	**Osmolality (mOsm/kg)**	**pH**	**Total protein (μg/μl)**	**Sterility**	**Endotoxin**
1	294	7.6	41	No growth	0.04
2	312	7.8	44	No growth	0.06
3	288	7.7	42	No growth	0.06
4	262	7.5	37	No growth	0.02
5	283	7.4	42	No growth	0.03
6	262	7.6	42	No growth	0.02

*There were no significant differences between blastocyst rate and morphological scoring obtained from the control vs. 10% HUF embryos of each patient (Table 4B). Five of these women achieved a successful pregnancy (Table 4C). Two of the babies came from embryos cultured with the uterine fluid of the mother, while the other three were produced under control conditions*.

**Table 4B:** Blastocyst rate and embryo quality according to morphological criteria described previously (Staicu et al., 2021) of control vs. 10% HUF embryos of each patient who accepted to participate in the proof-of-concept NCT03436758QC and fulfilled all the inclusion criteria, namely, the retrieval of at least eight metaphase II oocytes, and transfer of their embryos.

**PATIENT**	**OOCYTES**	**Exp. Group**	**MII**	**CLEAVAGE (%, N)**	**BLASTOCYST RATE (%, N)**	**Morphological scoring at day 5/6 of embryo culture**
1	13	HUF	6	67 (*N* = 4)	100 (*N* = 4)	2B /2C
		Ctrl	6	67 (*N* = 4)	100 (*N* = 4)	2A /2B
2	10	HUF	5	40 (*N* = 2)	50 (*N* = 1)	1B
		Ctrl	5	80 (*N* = 4)	25 (*N* = 1)	D
3	17	HUF	8	75 (*N* = 6)	33 (*N* = 2)	1A/1C
		Ctrl	7	100 (*N* = 7)	29 (*N* = 2)	1C /1C
4	17	HUF	8	75 (*N* = 6)	33 (*N* = 2)	1B/1C
		Ctrl	7	86 (*N* = 6)	83 (*N* = 5)	2A/3B
5	25	HUF	7	86 (*N* = 6)	17 (*N* = 1)	1B
		Ctrl	7	86 (*N* = 6)	17 (*N* = 1)	1C
6	19	HUF	8	75 (*N* = 6)	17 (*N* = 1)	1A
		Ctrl	7	100 (*N* = 7)	43 (*N* = 3)	2A/1C

**Table 4C:** Clinical outcomes of embryo transfer to each receptor. Each embryo was blindly selected according to the porphological criteria described previously (Staicu et al., 2021).

**Patient**	**Embryo transfer**			**Weeks gestation**	**Childbirth**	**Newborn**
	**Exp. group**	**MorphologicalScore**	**Result**			
1	CONTROL	1A	Pregnancy	38 + 6	Vaginal	♂
2	HUF	1B	Pregnancy	41 + 0	Vaginal	♂
3	HUF	1A	Abortion			
4	CONTROL	1A	Pregnancy	40 + 1	Caesarean	♂
5	HUF	1B	Pregnancy	40 + 2	Caesarean	♀
6	CONTROL	1A	Pregnancy	37 + 1	Vaginal	♂

## Discussion

For years, different strategies have been developed to improve the composition of the embryo culture media. The protein supplements currently used are scarce or poorly defined, and thus represent a gap in control of quality in the clinical embryology laboratory (Morbeck et al., 2014b). Mimicking as much as possible the physiological fluids of the female reproductive tract is logical and for instance, some of the proteins identified in these fluids have been added to the media, trying to characterize their possible benefits on the embryos, such as GMC-SF (Granulocyte-macrophage colony-stimulating factor) in humans (Sjöblom et al., 1999) or OVGP1 (Oviduct-specific glycoprotein 1) in livestock species (Coy et al., 2008). However, the high cost of their synthesis in the laboratory, the realization that the whole list of proteins in the fluids is enormous and therefore unattainable (Canha-Gouveia et al., 2019), and the observed inefficacy of their individualized addition in biological or clinical assays for GMC-SF (Ziebe et al., 2013) and OVGP1 (Algarra et al., 2018) have led to a conclusion that adding RFs as supplements to culture media should be a more efficient approach to palliate the short and long-term undesirable effects of ART (Coy and Yanagimachi, 2015). In this study, we aimed to evaluate the feasibility of (i) collecting HRF *in vivo* and *ex vivo*; (ii) developing protocols to guarantee the biosecurity of such fluids, allowing at the same time the storage of the maximum possible volume at a biobank, and (iii) enabling the use of HRF in the routine work of a fertility clinic.

The first conclusion we reached was that there are no suitable devices in the market to collect uterine fluid trans-vaginally in oocyte donors without damaging the endometrium since the negative pressure exerted by aspiration plungers in commercial devices always causes tissue injury. For this reason, in parallel with this study, a new device has been designed to allow the collection of fluid by capillarity, which is currently being tested (Canha-Gouveia and Latorre, 2019). The preliminary data showed that the fluid collected with this new prototype contains less blood and cellular debris than any other commercial device tested before, therefore offering a promising solution for the future collection of fluid in donors or patients.

As for protocols to establish physiological ranges for the safe use of the fluids, we show that a volume of 30 μl is enough to get reliable data for pH, osmolality, protein and albumin concentration, and BEA. This is of enormous importance for the future use of tested batches of HRF stored at a biobank. In this study, although we have checked fluids from women of different ages and physiological status, in the future, the final objective would be the use of only *in vivo*-collected HUFs from young donors (~20–30 years old), with proven fertility from previous oocyte donations, pooled in homogeneous batches, fully QC tested, and stored at a biobank. Regarding the HOFs, and due to the impossibility of realizing *in vivo* collections, the proposal is to use only those collected from healthy women of proven fertility who are attending hospitals to undergo a planned bilateral salpingectomy by laparoscopy as a contraceptive method. By introducing the collection of these fluids to a particular hospital of any geographical region as a routine, the amount that can be stored at a biobank would be more than enough to supplement all the culture media used by patients under IVF cycle conditions in the population covered by that hospital, bearing in mind that only 0.1–0.5 μl of fluid is necessary per embryo (culture drops are usually between 10 and 50 μl). Therefore, every 100 patients, properly scheduled at the appropriate phase of the cycle, would provide enough HRFs to culture ~11,500–23,000 embryos.

Another crucial point of discussion is the fact that reproductive fluids are a source of variability and that their addition to the culture media represents the loss of the so-called chemical definition. In the opinion of the authors, any biological process is, by definition, subjected to individual variability. Thus, standardized cycles in ART will never be reached just because each patient responds differently to hormone stimulation, possesses gametes with different genetics, and embryologists and physicians participating in the whole process are susceptible to errors. Needless to say, commercial media are also subjected to human intervention during their preparation, and human recombinant albumin, hyaluronic, and some other molecules included in the media cannot be considered as chemically defined. Finally, and despite the global tendency to use protein-free media, the benefit that such media provide to the embryo vs. future health repercussions of growing with a medium lacking hundreds of natural components should be considered, since differences between *in vivo-* and *in vitro*-grown embryos have become evident (Munné et al., 2019). As a useful comparison, one could try to imagine the world if the use of blood for transfusions was prohibited for the sake of “standardization,” and chemically defined blood was manufactured and used instead.

Most of the physicochemical parameters assessed in the different types of HRF in this study were within the physiological ranges. For osmolality, less variability was observed between the *in vivo* samples, and a higher variability was observed in the HOFs. Osmolality has been established around 250–300 mOsmol for most commercially available ART culture media (Gruber and Klein, 2011). However, these values may not be physiological, since the osmotic pressure of oviduct fluids in mice was reported to be higher than 360 mOsmol (Borland et al., 1980). Other studies have shown similar osmolality levels between HUF and serum and the range was below the data for HOFs (Casslen and Nilsson, 1984). This gave us the confidence to suggest that HOFs could frequently have higher osmolality than HUFs, and that this fact should be considered in the elaboration of commercial media.

Regarding pH, again, less variability was observed between the *in vivo* samples. According to Phillips et al. (2000), the pH range for embryo culture media may be set between 7.4 and 7.2, but most laboratories usually decrease these figures during the culture of cleavage stage embryos and increase them during the morula and blastocyst stages (Swain, 2012). From the data of this study, it seems clear that the limits could be established higher (7.4–8) for embryo culture media, and that IVF media should be set at a lower pH. Although the pH in the female reproductive tract is graduated, with the lowest pH in the vagina (~pH 4.42) increasing toward the fallopian tubes (~pH 7.94) (Ng et al., 2000), it is also known that the variation in these values could reflect the variation in the site-specific microbiome and acid-base buffering at the tissue/cellular level and responses to menstrual cycle (Chen et al., 2017). Consequently, it is logical that the shortest interval of values, not only for pH but also for osmolality, was found in HUF *in vivo*, whose collection was performed in oocyte donors at the same phase of the menstrual cycle (postovulatory), while the other RFs were collected from women in different phases of the menstrual cycle.

One of the most standardized and informative parameters that can be assessed in any biological fluid is total protein concentration, and, particularly, in the embryo culture medium, albumin is of striking importance (Morbeck et al., 2014a). Albumin has a major role as a carrier for lipids, hormones, and minerals (Lane and Gardner, 2007). However, it can also be associated with globulins, and studies are suggesting that supplementation of human embryo culture media with 16% globulins in addition to 84% albumin results in an overall increase in implantation and live birth rates (Meintjes et al., 2009). This observation supports the idea of the authors and others about the need for proteins in *in vitro* embryo culture media (Itze-Mayrhofer and Brem, 2020). It is known that albumin is the major protein in RFs as well as in blood serum; therefore, total protein concentration should be correlated with albumin concentration for each sample. That is why the selected method in this study was the total protein measurement instead of the expensive human albumin binding assay. For total protein concentration, we found significant differences between HOFs and HUFs, both *in vivo* and *ex vivo* (*p* < 0.05), and blood contamination was not the reason for such differences, as the data of hemoglobin concentration demonstrate. A previous study has also shown a low total protein concentration in oviductal fluids (Canha-Gouveia et al., 2019), so this should be considered for the different formulations of IVF media vs. embryo culture media. As explained above, and although chemically defined media are considered safe, the lack of proteins could have an impact on the epigenetic reprogramming of the embryo and even on fetal and offspring development (Blake et al., 2002; Chronopoulou and Harper, 2015). For that reason, once again, we consider it important to include proteins in the culture media.

The mouse embryonic assay (Ackerman et al., 1984, 1985; Byers et al., 2006) is commonly performed by companies manufacturing culture media. Although mice are still the most popular species used to this end, we consider their use unnecessary, and contrary to the 3 Rs recommendations from Russell and Burch's principle (Russell and Burch, 1959; Kirk, 2018); in other words, the sacrifice of animals with the only purpose of testing the toxicity of fluids in a biological assay should be avoided. Instead, livestock species offer an alternative option as animal models to check the safety of any reagent added to *in vitro* embryo culture media, since the availability of gametes in slaughterhouses is practically unlimited. In bovine species, the average percentage of bovine embryos at the two-cell stage on day 2 of embryo culture is around 70%, and the percentage of embryos expanded to blastocyst is close to 30% (Lonergan and Fair, 2014). Although these values are lower than ones usually shown by MEA, this can be explained because the strain of mouse embryos commonly used for quality control in IVF laboratories almost always have > 90% blastocyst development, possibly due to improved culture conditions, which hinder the detection of negative effects of media or contact materials that would otherwise affect human embryo development (Kaskar et al., 2019). The BEA results in this study showed that this can be a suitable test to detect alteration in the functionality of HRFs that could be toxic for embryos. Even with fluids falling in the ranges we have determined as physiological for osmolality, pH, endotoxins, etc., and apparently suitable, the BEA test can detect other unrecognized factors in the fluids affecting gamete interactions or the embryo development, as it was the case. Therefore, BEA is proposed as a suitable test to replace MEA for this type of QC to avoid unnecessary animal sacrifices and because cow, at the reproductive level, is more like humans than mice (Abedal-Majed and Cupp, 2019).

Finally, we examined the option of introducing the HRF in the routine of a fertility clinic, starting with an assay adding fluids of patients receiving their embryos, as a preliminary proof of concept before starting clinical studies with a larger sample size or use of heterologous HRF. This study had two main limiting factors: on the one hand, the collected volume of fluids, since the fluid of the patient must be used for all QC analyses and embryo culture, and on the other hand, the number and quality of harvested oocytes. Although all the women who participated in this study were under 39 years old and had no diagnosis of endometriosis or uterine pathology, medical history of repeated abortion or implantation failure or abnormal karyotype, it was not possible to retrieve at least eight metaphase II oocytes from eight women who previously accepted to participate in the proof-of-concept NCT03436758QC. For this reason, they were not included in this pilot study. The other six women fulfilled all the inclusion and exclusion criteria, and their fluids fitted all the parameters established previously in QC sheet for human reproductive fluids (RFs) (Table 4A). Five of these women achieved a successful pregnancy (Table 4C). Two of the babies came from embryos cultured with the uterine fluid of the mother, while the other three were produced under control conditions. With this small number of patients involved and consequent pregnancies, we cannot confirm that the supplementation of embryo culture media with RFs could be a feasible strategy to mimic as much as possible the natural environment where humans develop, as has been previously demonstrated in other animal species. However, the birth of the two babies is a very promising event. Funding and logistic conditions are needed to extend this study to definitively clarify the feasibility of the strategy. Furthermore, in further studies with a higher number of patients, it will be pertinent to perform a preimplantation genetic screening of embryos produced with and without HUFs to assess their effect at the molecular level. Afterward, the feasibility of the use of HRFs must be tested in batches of proven quality from heterologous donors to examine their effect on embryo quality, embryo implantation rate, and short and long-term health of newborns.

In conclusion, this study standardizes the characteristics that collected HRF must exhibit to be incorporated into embryo culture media. A fully characterized biobank collection of HRFs following the quality control protocols described in this study has been created at Biobanc-Mur and will be crucial for pertinent future clinical trials to demonstrate the benefits of HRFs with a suitable sample size.

## Data Availability Statement

The original contributions presented in the study are included in the article, further inquiries can be directed to the corresponding author.

## Ethics Statement

This study was approved by the Ethics Research Committee (CEIC) of Clinical University Hospital “Virgen de la Arrixaca” (HCUVA), Murcia, Spain (Approval No. EST: 04/16). All the women who accepted to participate in this study provided their written informed consent. The proof of concept “Validation of Addition of Uterine Fluid to Human Embryo Culture Medium” –1607-MUR-055-JL/ was approved on 08/21/2019 and registered on ClinicalTrials.gov of the United States National Library of Medicine with the identifier NCT03436758.

## Author Contributions

PC conceived the idea. PC and AC-G designed the study, wrote the first manuscript draft, and analyzed the data. AC-G, EP-O, and CS-Ú performed the analyses and data collection. MP-S and MS-F provided the ex vivo HUF and HOF samples at the Hospital. JL, JM-S and MM provided the in vivo HUF samples at the infertility clinic. The manuscript was written through contributions of all authors. All authors have given approval to the final version of the manuscript. All authors contributed to the article and approved the submitted version.

## Conflict of Interest

The authors declare that the research was conducted in the absence of any commercial or financial relationships that could be construed as a potential conflict of interest. The reviewer MH declared a past collaboration with one of the authors PC.

## Publisher's Note

All claims expressed in this article are solely those of the authors and do not necessarily represent those of their affiliated organizations, or those of the publisher, the editors and the reviewers. Any product that may be evaluated in this article, or claim that may be made by its manufacturer, is not guaranteed or endorsed by the publisher.
